# Relationship between depression, anxiety, stress, and SARS-CoV-2 infection: a longitudinal study

**DOI:** 10.3389/fpsyg.2023.1116566

**Published:** 2023-04-27

**Authors:** Dietmar Ausserhofer, Angelika Mahlknecht, Adolf Engl, Giuliano Piccoliori, Gernot Pfitscher, Philipp Silbernagl, Francesca Giacomoni, Roger Pycha, Stefano Lombardo, Timon Gärtner, Michael Mian, Horand Meier, Christian J. Wiedermann, Roland Keim

**Affiliations:** ^1^Institute of General Practice and Public Health, College of Health Care-Professions Claudiana, Bolzano-Bozen, Italy; ^2^Claudiana Research, College of Health Care-Professions Claudiana, Bolzano-Bozen, Italy; ^3^Hospital of Bressanone-Brixen (SABES-ASDAA), Teaching Hospital of the Paracelsus Medical Private University (PMU), Bressanone-Brixen, Italy; ^4^Statistical Institute of the Province of Bolzano (ASTAT), Bolzano-Bozen, Italy; ^5^Innovation, Research and Teaching Service (SABES-ASDAA), Teaching Hospital of the Paracelsus Medical Private University (PMU), Bolzano-Bozen, Italy; ^6^Clinical Governance Unit, Administration of the Autonomous Province of Bolzano-Bozen, Bolzano-Bozen, Italy; ^7^Department of Public Health, Medical Decision Making and Health Technology Assessment, University of Health Sciences, Medical Informatics and Technology, Hall in Tirol, Austria

**Keywords:** COVID-19, SARS-CoV-2, emotional burden, depression, stress, anxiety, longitudinal study

## Abstract

**Objectives:**

We aimed to (1) describe the course of the emotional burden (i.e., depression, anxiety, and stress) in a general population sample during the coronavirus pandemic in 2020 and 2021 and (2) explore the association between emotional burden and a serologically proven infection with SARS-CoV-2.

**Study design:**

This longitudinal study involved a sample of community-dwelling persons aged ≥14 years from the general population of South Tyrol (Province of Bolzano-Bozen, Northern Italy). Data were collected at two stages over a 1-year period in 2020 and 2021.

**Methods:**

Persons were invited to participate in a survey on socio-demographic, health-related and psychosocial variables (e.g., age, chronic diseases, Depression Anxiety Stress Scale, DASS-21), as well as in the serological testing for of SARS-CoV-2-specific immunoglobulins.

**Results:**

In 2020, 855 (23.8%) out of 3,600 persons participated; in 2021, 305 (35.7%) out of 855 were tested again. We observed a statistically significant decrease in mean DASS-21 scores for depression, stress, and total scores between 2020 and 2021, yet not for anxiety. Persons with a confirmed SARS-CoV-2-infection between the first and second data collection exhibited increased emotional burden compared to those without SARS-CoV-2-infection. The odds of participants with a self-reported diagnosis of mental disorder for future infection with SARS-CoV-2 was almost four times higher than that of participants without mental disorders (OR:3.75; 95%CI:1.79-7.83).

**Conclusion:**

Our findings support to the hypothesis of a psycho-neuroendocrine-immune interplay in COVID-19. Further research is necessary to explore the mechanisms underlying the interplay between mental health and SARS-CoV-2 infections.

## 1. Introduction

The association between an infection with severe acute respiratory syndrome coronavirus 2 (SARS-CoV-2) and emotional disorders such as depression and anxiety post COVID has been shown in several studies, including inpatients (Daher et al., 2021; Tomasoni et al., 2021) and outpatients (Magnúsdóttir et al., 2022), at different time points, i.e., weeks (Halpin et al., 2021; Méndez et al., 2021) and even months after the infection (Hüfner et al., 2022; Kim et al., 2022). A systematic review of eight studies on depressive symptoms 12 weeks after a SARS-CoV-2-infection estimated a prevalence between 11 and 28% (Renaud-Charest et al., 2021). The relatively wide range can be explained by the use of different instruments, differences in acute physical symptom severity, differences in sociocultural background, and/or different recruitment methods (Thye et al., 2022). Moreover, there is increasing evidence on the association between the COVID-19 pandemic and affective symptoms and psychological distress across countries (Brailovskaia et al., 2021), as well as fatigue, loneliness and violent behaviours (Emmelkamp, 2021; Mansueto et al., 2021) and dysfunctional coping strategies, i.e., covid anxiety syndrome (Mansueto et al., 2022).

The high amount of emotional distress in patients with a prior SARS-CoV-2-infection could be due to the infection itself, but we cannot dismiss the possibility that subjects with high depression, anxiety, or stress scores may have had elevated levels before the infection, which in turn might have increased the likelihood of an infection with SARS-CoV-2. Neuroendocrine-immune mechanisms might boost the risk of a SARS-CoV-2 infection and have a negative impact on the course of the following coronavirus disease 19 (COVID-19). However, epidemiological data on the hypothesized interplay between emotional distress and an infection with SARS-CoV-2 are largely missing (Peters et al., 2021). Experimental studies have revealed that pre-existing emotional burden, such as stress or the feeling of loneliness, entails a risk of viral infection and may negatively impact the course of infectious diseases (Cohen et al., 1991; Cole et al., 2011; Cohen et al., 2015; LeRoy et al., 2017; Cohen, 2021). Similarly, high mortality rates after SARS-CoV-2 infection have been observed in patients with schizophrenia (Nemani et al., 2021). Interactions between the brain and immune system, already known from psycho-neuro-immunological research, have been observed in the COVID-19 pandemic (Mehta et al., 2021).

Yet, most studies on the association between COVID-19 and the emotional burden relied on self-reports of prior infection with the SARS-CoV-2 virus only, thus excluding asymptomatic or not diagnosed patients. More importantly, they applied a cross-sectional design, which poses methodological problems in interpreting the results (Renaud-Charest et al., 2021; Hüfner et al., 2022). As studies monitoring immunological and psychological parameters have been urgently called for (Rademacher et al., 2021), longitudinal studies are needed to observe over time changes in emotional burden and the occurrence of SARS-CoV-2 infection to provide more robust evidence on the direction of the association between psychological burden and COVID-19. To explore the association between emotional burden and infection with the SARS-CoV-2 virus, we conducted a longitudinal study over 12 months, measuring depression, anxiety, and stress together with serological testing for an infection with the SARS-CoV-2 virus at two time points during the pandemic.

### 1.1. Objectives

Therefore, we aimed to describe the course of the emotional burden (i.e., depression, anxiety, and stress) in a general population sample during the coronavirus pandemic in 2020 and 2021; and to explore the association between emotional burden and infection with SARS-CoV-2.

## 2. Methods

### 2.1. Study design

This longitudinal study involved a cohort of persons participating in two representative cross-sectional seroprevalence studies in the general population of South Tyrol (Province of Bolzano, Northern Italy). The first data collection took place between July 6 and July 27, 2020, and the second between June 16 and July 9, 2021, revealing an infection-induced seroprevalence of 2.9 and 27.9%, respectively (ASTAT, 2020).

### 2.2. Sample and setting

For the first data collection between June 1 and July 31, 2020 (t_1_), all community-dwelling persons aged >5 years were eligible to participate in the seroprevalence study. South Tyrol, the Autonomous Province of Bolzano, is part of the Trentino–Alto Adige region in Italy, next to Austria (total population, 534.912), with approximately 70% German-speaking, 25% Italian-speaking inhabitants, and 5% other languages. The Statistical Institute of the Province of Bolzano (Istituto Provinciale di Statistica – Landesinstitut für Statistik) randomly selected study participants from the register of the current resident population in the whole province (ASTAT, 2020). The study sample was stratified by age, sex, and residency. The necessary sample size of 3,600 persons was calculated by the statistical institute based on the current rate of persons testing positive for SARS-CoV-2 and an estimated number of undetected cases. In total, 855 persons aged ≥14 years participated in the first data collection (t_1_), i.e., survey and serological detection of SARS-CoV-2 infection.

In the second data collection between June 1 and July 31, 2021 (t_2_), all participants of the first data collection in 2020 (*n* = 855) were eligible for participation. Selected individuals were invited to participate by mail. The invitation letter was sent in German and Italian language and contained (i) all relevant information about the study procedures; (ii) date, time, and location of planned sample-taking (swab test and blood test); and (iii) the URL of the online questionnaire with a personalized password (see below). Informed consent was delivered to and signed by the study participants directly onsite before taking biological samples. From the 855 persons participating in the first data collection 2020 (t_1_), a total of 305 persons participated in the second data collection in 2021 (t_2_).

### 2.3. Variables and measures

For this study, data from a participant survey and from the serological test for SARS-CoV-2-specific immunoglobulins were collected and analyzed. To access the online questionnaire at both time points, a personalized password was delivered with a written invitation letter (see above), which served as a pseudonymization codex throughout the study period. This codex was also applied to the corresponding biological samples to enable a link between the questionnaire results and biological sample results. The online survey was created using SoSci Survey.^1^ The questionnaire was additionally provided in paper form at the onsite testing points for those participants who had not completed the online questionnaire.

#### 2.3.1. Participant survey

The *socio-demographic and epidemiologic part* of the questionnaire was based on previous national investigations from the Italian Statistical Institute (ISTAT, 2020) and assessed the following information:

- Socio-demographic data: age, sex, place of birth, native tongue, nationality, residence, level of education, current working situation;
- Health-related questions: current health status, current or former occurrence of COVID-19-like symptoms, chronic conditions and pre-existing health problems including mental health, worsening of pre-existing health issues during the pandemic, current medication, previous SARS-CoV-2 swab or antibody test and testing result, and home isolation or hospitalization in case of COVID-19 infection.

The *psychologic part* assessed behavioral habits and burdens during the pandemic, as well as emotional risk factors with potential negative impact on the immune system. Depression, anxiety, and stress were measured with the Depression Anxiety Stress Scale (DASS-21) in the German (Nilges and Essau, 2015) and Italian (Bottesi et al., 2015) versions. The scale has shown good psychometric properties (Antony et al., 1998). The DASS-21 consists of 21 items, with seven items for each subscale. Subjects were asked to score each item on a 4-point Likert scale ranging from 0 (‘did not apply to me at all’) to 3 (‘applied to me very much’). According to the classification provided from S Lovibond and P Lovibond (Lovibond and Lovibond, 1993), we calculated the percentages of as “high” or “severe” emotional burden according to the following cut-offs: DASS-21-total sore (≥60 points), depression (≥21 points), anxiety (≥15 points) and stress (≥26 points).

As the emotional burden, including depression is strongly associated with fatigue, we aimed to control for this potential confounding variable and measured fatigue using the Fatigue Severity Scale (FSS) (Krupp et al., 1989). This 9 items questionnaire investigates the severity of fatigue in various situations during the previous week on a 7-point Likert scale, where 1 indicates strong disagreement and 7 indicates strong agreement. The final score is defined as the mean value. The scale was validated with different patient cohorts both in the German (Valko et al., 2008) and the Italian language (Ottonello et al., 2016) showing good psychometric properties.

As social behavior was greatly affected during the pandemic (e.g., lockdown, quarantine), which might be associated with both, emotional burden and the occurrence of SARS-CoV-2 infection, we considered social network size and diversity as control variables, measured with the social network index (SNI) (Cohen et al., 1997). More diverse social network was associated in prior research with greater resistance against upper respiratory illness (Cohen et al., 1997). The SNI measures participation in 12 types of social relationships (spouse, parents, parents-in-law, children, other close family members, close neighbors, friends, workmates, schoolmates, fellow volunteers, members of groups without religious affiliation, and religious groups) and the number of persons in contact per relationship. One point was assigned for each type of relationship (highest possible score = 12) when respondents indicated that they spoke (in person or over the phone) to persons in that relationship at least once every 2 weeks. Moreover, participants indicated the number of people for each relationships to which they spoke at least once every 2 weeks to calculate the total number of network members for each participant (Cohen et al., 1997).

Sleep is closely linked to mental health and well-being. While poor sleep quality and sleep disturbances have been shown to be associated with a range of mental health problems (Blackwelder et al., 2021), as well the stress, anxiety and depression during the pandemic (Franceschini et al., 2020), improved sleep quality leads to better mental health outcomes (Scott et al., 2021). We aimed to control for sleep quality in our analyses and measured sleep duration as the self-reported mean hours of sleep per night during the last month, and sleep quality was assessed using a single item on a 3-point Likert scale (i.e., poor, moderate, or very good).

#### 2.3.2. Detection of SARS-CoV-2 infection

The testing procedures and analyses of the biological samples were planned and coordinated by the local representative of the National Health Service (Servizio Sanitario Nazionale) in collaboration with the research team. The personnel who conducted the on-site testing received instructions and a checklist of all procedures from the study group. For the detection of SARS-CoV-2-specific immunoglobulins, blood tests for the quantitative determination of IgM and IgG antibodies (without differentiation) based on the nucleocapsid (N) antigen by means of Elecsys® Anti-SARS-CoV-2 assay (Roche Diagnostics) were applied, considering a cut-off index for a SARS-CoV-2 infection ≥1.0.^2^ The biological samples were tagged with the individual pseudonymization codex (corresponding to the codex of the questionnaire) and transported to the analysis laboratory of the Central Hospital of Bolzano – Bozen. Serological results were communicated to the study participants according to the standard operating procedures and transferred to the research team in a pseudonymized form at the end of data collection.

### 2.4. Ethical aspects

The study was approved by the responsible ethics committee of Bolzano on 28/05/2020 (approval number 52-2020) and the amendment on 22/04/21 (approval number 51-2021). All study procedures were in accordance with the 1964 Helsinki Declaration and its amendments, European Union General Data Protection Regulation (679/2016), and Italian Data Protection Law (196/2003). All study participants provided written informed consent before their inclusion.

### 2.5. Statistical analyses

Descriptive statistics (e.g., frequencies, means, and SDs) were calculated to describe the measured variables. To explore differences on categorical and non-normally distributed continuous variables between the characteristics of participants in t_1_ and t_2_ and participants in t_1_ only, chi-square and Mann–Whitney U tests were performed. Student’s t-tests were applied for continuous variables that were normally distributed according to Kolmogorov–Smirnov test to explore differences in emotional burden (DASS-21 scale) between 2020 and 2021 and between persons with and without confirmed SARS-CoV-2 infection for participants in t_1_ and t_2_. To explore the associations between emotional burden and SARS-CoV-2 infection, regression analyses were performed. First, four multiple linear regression models were computed with confirmed SARS-CoV-2 infection and other sociodemographic variables as independent variables, and changes in depression, anxiety, and stress levels (DASS-21 total score and depression, stress, and anxiety subscales) between 2020 and 2021 as dependent variables. Similarly, a binary logistic regression model was used to explore the association between depression, anxiety, and stress in 2020 (independent variables) and the confirmed SARS-CoV-2 infection in 2021 (dependent variable). Major potentially confounding variables, including psychosocial and health-related factors (e.g., social network, sleep quality, and fatigue), were included in the regression analyses. All data analyses were performed using the IBM SPSS Statistics (version 27). A *p* value of less than 0.05 was considered significant.

## 3. Results

In the 2020 data collection, out of a total of 3,600 invitations, 855 persons (23.8%) participated in both the survey and serological detection of SARS-CoV-2-specific immunoglobulins. In the 2021 data collection, 305 (35.7%) of the 855 persons participated. Table 1 shows the characteristics of the study participants at both time points. Compared to the first data collection in 2021, women, persons aged 45 or older and Italian citizens were more likely to participate in the second data collection.

**Table 1:** Characteristics of study participants in 2020 (*n* = 855) and comparison between study participants in 2020 (t_1_) and 2021 (t_2_) (*n* = 305) and study participants in 2020 (t_1_) only (*n* = 550).

Variables	Total sample in 2020 (*n* = 855)		Study participants in 2020 (t_1_) and 2021 (t_2_) (*n* = 305)		Study participants in in 2020 (t_1_) only (*n* = 550)		*p* ^#^
Sex, *n* – %							
Female	448	52.4	174	57.0	251	49.0	0.026*
Male	407	47.6	131	43.0	261	51.0	
Age, *n* – %							
14–20 years	33	4,0	6	2.0	26	5.1	<0.001*
21–44 years	263	32.0	72	23.6	189	36.9	
45–65 years	341	41.5	152	49.8	189	36.9	
66 years and older	184	22.4	75	24.6	108	21.1	
Native tongue, *n* – %							
German	534	71.3	199	71.8	312	71.4	0.305
Italian	160	21.4	64	23.1	88	20.1	
Ladin	23	3.1	7	2.5	16	3.7	
Other	32	4.3	7	2.5	21	4.8	
Citizenship, *n* – %							
Italian	812	95.0	301	98.7	479	93.6	0.001*
other	43	5.0	4	1.3	33	6.4	
Educational level, *n* – %							
Below Highschool	384	51.3	137	49.5	225	51.5	0.597
Highschool or higher	365	48.7	140	50.5	212	48.5	
Employment, *n* – %							
Employed (including housewife)	545	73.0	202	72.9	318	73.1	0.243
Unemployed/job seekers	15	2.0	2	0.7	12	2.8	
Retired	150	20.1	60	21.7	83	19.1	
Student	37	5.0	13	4.7	22	5.1	
Household, *n* – %							
Living alone	105	12.8	273	89.5	439	85.7	0.120
Living with partner/family	715	87.2	32	10.5	73	14.3	
Social Network							
Number of people, Median – IQR	13	12	13	12	12	11	0.170
Network diversity, Median – IQR	7	3	7	3	7	3	0.181
Overall good or very good health status, *n* – %	673	89.9	253	91.3	391	89.5	0.415
1 or more chronic diseases	59	19.3					
Diagnosed mental disorder (e.g., depression and/or anxiety)	26	3.0	5	1.6	19	3.7	0.090
Sleep hours, Median – IQR	7	1	7	1	7	1	0.279
Sleep quality							
Poor	69	8.5	27	8.3	42	8.3	0.899
Medium	495	60.9	187	60.7	308	60.7	
Very Good	249	30.6	90	31.0	157	31.0	
Confirmed infection with SARS-CoV-2							
Yes	31	3.7	60	20.0	NA	NA	
No	800	96.3	240	80.0	NA	NA	
Vaccinated against COVID-19 in 2021							
Yes	NA	NA	246	80.7	NA	NA	
No	NA	NA	59	19.3	NA	NA	
DASS-21 Depression, Mean – SD	3.8	5.8	4.2	6.5	3.6	5.5	0.204
DASS-21 Anxiety, Mean – SD	2.6	4.4	2.8	4.6	2.5	4.2	0.334
DASS-21 Stress, Mean – SD	6.1	6.9	6.4	7.5	5.9	6.6	0.324
DASS-21 Total, Mean – SD	12.5	15.4	13.4	16.8	12.1	14.6	0.232

# Chi-square test, Mann–Whitney-U test or Student’s *t*-test, NA, not assessed. *Statistically significant.

### 3.1. Emotional burden in 2020 and 2021

Among the cohort members, less than 5% had high or very high DASS-21 depression (2020:3.0% vs. 2021:4.0%), anxiety (2020:3.3% vs. 2021:3.6%), stress (2020:3.9% vs. 2021:2.2%), and total scores (2020:3.3% vs. 2021:2.6%) at both time points. As summarized in Table 2, we observed a statistically significant decrease in the mean DASS-21 depression, stress, and total scores between 2020 and 2021, but not for anxiety. Participants with a confirmed SARS-CoV-2 infection had significantly higher DASS-21 depression, anxiety, stress, and total scores in 2021 than those without confirmed SARS-CoV-2 infection (see Table 3).

**Table 2:** Summary statistics for the DASS-21 study participants (t_1_ and t_2_) and comparison between 2020 and 2021 (*n* = 305).

Variables	2020		2021		*T(p)* ^#^
	Mean	SD	Mean	SD	
DASS-21 Depression (0–42)	4.19	6.49	3.44	6.58	−2.21(0.028)*
DASS-21 Anxiety (0–42)	2.87	4.64	2.48	4.63	−1.50(0.148)
DASS-21 Stress (0–42)	6.48	7.46	5.13	7.10	−3.64(<0.001)*
DASS-21 Total (0–126)	13.54	16.82	11.04	16.79	−2.95(0.003)*

Scores on the DASS-21 items were multiplied by 2 to calculate the subscale and total scores, producing a maximum of 42 points and 126 points, respectively.

# Student’s *t*-tests. *Statistically significant.

**Table 3:** Comparing depression, anxiety and stress (DASS-21) for study participants (t_1_ and t_2_) between those with and without confirmed SARS-CoV-2 infection in 2021 (*n* = 305).

Variables	SARS-CoV-2 infection (positive anti-nucleocapsid test) *n* = 61		No SARS-CoV-2 infection (negative anti-nucleocapsid test) *n* = 241		*T(p)* ^#^
	Mean	SD	Mean	SD	
DASS-21 Depression (0–42)	5.68	7.81	2.82	6.22	2.58(0.012)*
DASS-21 Anxiety (0–42)	3.79	5.58	2.16	4.41	2.06(0.043)*
DASS-21 Stress (0–42)	7.16	7.89	4.66	6.93	2.35(0.021)*
DASS-21 Total (0–126)	16.63	19.22	9.64	15.98	2.51(0.014)*

Scores on the DASS-21 items were multiplied by 2 to calculate the subscale and total scores, producing a maximum of 42 points and 126 points, respectively.

# Student’s *t*-tests. *Statistically significant.

In the subgroup of 67 subjects that in 2021 were above the cut-off for normal DASS-21 depression scores (>9 points), 34 out of 39 subjects with a confirmed SARS-CoV-2 infection between 2020 and 2021 had shown normal DASS-21 depression scores in 2020, while this was the case in only 8 out of 28 subjects without SARS-CoV-2 infection. Thus, the risk of developing relevant depressive symptoms after SARS-CoV-2 infection for unremarkable subjects was 3.05 times higher compared to non-infected subjects.

### 3.2. Association between SARS-CoV-2 infection and emotional burden

As reported in Tables 4–6, evidence of SARS-CoV-2 infection was associated with an increase in depression, anxiety, and stress levels, as well as with an overall worsening of emotional well-being (DASS-21-total score; Table 7). More precisely, in subjects tested positive for SARS-CoV-2 specific antibodies we observed a higher increase in depression, anxiety, and stress levels compared to those without SARS-CoV-2 infection after adjusting for major socio-demographic and health-related (e.g., age, chronic diseases, and vaccination against COVID-19), as well as psychosocial and individual factors (e.g., social network, sleep quality, fatigue).

**Table 4:** Multiple linear regression analyses showing the association between confirmed SARS-CoV-2-infection and changes in depression (DASS-21 depression subscale) between 2020 and 2021 (*n* = 305).

Variables	*B*	Beta	SE	95%CI		*p*
				*LL*	*UL*	
SARS-CoV-2 infection (2021) (reference: no infection)	2.52	0.16	0.88	0.80	4.25	0.004*
Age (in years)	−0.02	−0.05	0.03	−0.08	0.04	0.488
Sex (reference: male)	1.34	0.10	0.76	−0.15	2.83	0.078
Native tongue (reference: German)	−0.15	−0.02	0.54	−1.21	0.90	0.778
Employment	0.49	0.08	0.41	−0.31	1.30	0.226
Educational level (reference: below high school)	−0.19	−0.02	0.74	−1.65	1.27	0.797
Vaccinated against COVID-19 (reference: yes)	0.09	0.01	0.77	−1.42	1.60	0.904
One or more chronic diseases (reference: no disease)	4.04	0.11	2.16	−0.23	8.31	0.063
Diagnosed mental disorder (reference: no mental disorder)	−0.25	−0.02	0.89	−2.00	1.49	0.774
Sleep – Hours	−0.20	−0.04	0.36	−0.91	0.50	0.572
Sleep–- Quality	0.37	0.03	0.69	−0.10	1.73	0.597
Social Network – Diversity	−0.30	−0.07	0.36	−1.02	0.42	0.414
Social Network – Number of people	0.09	0.12	0.06	−0.02	0.21	0.114
Fatigue (FSS)	1.24	0.27	0.32	0.62	1.87	<0.001*
DASS-21 Depression 2020	−1.28	−0.65	0.18	−1.63	−0.93	<0.001*
DASS-21 Anxiety 2020	0.19	0.07	0.22	−0.23	0.62	0.371
DASS-21 Stress 2020	0.05	0.03	0.16	−0.27	0.37	0.762

SE, standard error; CI, confidence interval; *LL*, lower limit; *UL*, upper limit. *R*^2^ = 0.38, *F*(17, 226) = 8.10, *p* < 0.001. *Statistically significant.

**Table 5:** Multiple linear regression analyses showing the association between confirmed SARS-CoV-2-infection and changes in anxiety (DASS-21 anxiety subscale) between 2020 and 2021 (*n* = 305).

Variables	*B*	Beta	SE	95%CI		*p*
				*LL*	*UL*	
SARS-CoV-2 infection (2021) (reference: no infection)	1.25	0.12	0.59	0.03	0.09	0.035*
Age (in years)	0.01	0.04	0.02	0.57	−0.03	0.570
Sex (reference: male)	0.10	0.01	0.51	0.85	−0.90	0.846
Native tongue (reference: German)	0.11	0.02	0.36	0.77	−0.60	0.769
Employment	0.29	0.07	0.27	0.28	−0.24	0.283
Educational level (reference: below high school)	0.32	0.04	0.49	0.52	−0.66	0.523
Vaccinated against COVID-19 (reference: yes)	−0.40	−0.04	0.51	0.44	−1.40	0.435
One or more chronic diseases (reference: no disease)	2.79	0.11	1.44	0.05	−0.05	0.054
Diagnosed mental disorder (reference: no mental disorder)	0.57	0.05	0.60	0.35	−0.62	0.345
Sleep – Hours	0.42	0.11	0.24	0.08	−0.06	0.084
Sleep - Quality	0.16	0.02	0.46	0.73	−0.75	0.734
Social Network – Diversity	−0.24	−0.08	0.24	0.33	−0.72	0.329
Social Network – Number of people	0.08	0.16	0.04	0.04	0.00	0.040*
Fatigue (FSS)	1.09	0.34	0.21	0.00	0.67	<0.001*
DASS-21 Depression 2020	−0.05	−0.04	0.12	0.67	−0.29	0.668
DASS-21 Anxiety 2020	−1.22	−0.67	0.14	0.00	−1.51	<0.001*
DASS-21 Stress 2020	0.11	0.09	0.11	0.31	−0.11	0.313

SE, standard error; CI, confidence interval; *LL*, lower limit; *UL*, upper limit. *R*^2^ = 0.41, *F*(17, 226) = 9.09, *p* < 0.001. *Statistically significant.

**Table 6:** Multiple linear regression analyses showing the association between confirmed SARS-CoV-2-infection and changes in stress (DASS-21 stress subscale) between 2020 and 2021 (*n* = 305).

Variables	*B*	Beta	SE	95%CI		*p*
				*LL*	*UL*	
SARS-CoV-2 infection (2021) (reference: no infection)	1.71	0.10	0.84	0.05	3.37	0.044*
Age (in years)	0.01	0.02	0.03	−0.04	0.06	0.712
Sex (reference: male)	1.54	0.11	0.73	0.11	2.97	0.035*
Native tongue (reference: German)	0.43	0.04	0.52	−0.59	1.44	0.407
Employment	0.35	0.05	0.39	−0.42	1.13	0.368
Educational level (reference: below high school)	−0.52	−0.04	0.71	−1.92	0.88	0.463
Vaccinated against COVID-19 (reference: yes)	−0.78	−0.06	0.73	−2.22	0.66	0.287
One or more chronic diseases (reference: no disease)	0.90	0.02	2.08	−3.20	5.01	0.665
Diagnosed mental disorder (reference: no mental disorder)	1.52	0.09	0.86	−0.17	3.20	0.077
Sleep – Hours	−0.24	−0.04	0.35	−0.92	0.44	0.489
Sleep – Quality	−0.34	−0.03	0.66	−1.64	0.97	0.611
Social Network – Diversity	0.04	0.01	0.35	−0.65	0.74	0.899
Social Network – Number of people	0.09	0.12	0.05	−0.02	0.20	0.094
Fatigue (FSS)	2.22	0.45	0.30	1.62	2.81	<0.001*
DASS-21 Depression 2020	−0.06	−0.03	0.17	−0.39	0.28	0.740
DASS-21 Anxiety 2020	0.10	0.04	0.21	−0.31	0.50	0.633
DASS-21 Stress 2020	−1.31	−0.71	0.16	−1.62	−1.01	<0.001*

SE, standard error; CI, confidence interval; *LL*, lower limit; *UL*, upper limit. *R*^2^ = 0.50, *F*(17, 228) = 13.20, *p* < 0.001. *Statistically significant.

**Table 7:** Multiple linear regression analyses showing the association between confirmed SARS-CoV-2-infection and changes in depression, anxiety and stress levels (DASS-21 total score) between 2020 and 2021 (*n* = 305).

Variables	*B*	Beta	SE	95%CI		***p***
				*LL*	*UL*	
SARS-CoV-2 infection (2021) (reference: no infection)	5.60	0.15	1.98	1.65	9.47	0.006*
Age (in years)	0.01	0.01	0.06	−0.12	0.13	0.924
Sex (reference: male)	2.96	0.10	1.72	−0.42	6.35	0.086
Native tongue (reference: German)	0.31	0.01	1.21	−2.07	2.69	0.798
Employment	1.22	0.08	0.92	−0.59	3.03	0.186
Educational level (reference: below high school)	−0.58	−0.02	1.68	−3.89	2.73	728
Vaccinated against COVID-19 (reference: yes)	−1.27	−0.04	1.74	−4.70	2.15	0.463
One or more chronic diseases (reference: no disease)	7.70	0.09	4.87	−1.89	17.30	0.115
Diagnosed mental disorder (reference: no mental disorder)	1.68	0.04	2.02	−2.31	5.66	0.408
Sleep – Hours	−0.07	−0.01	0.81	−1.67	1.54	0.933
Sleep – Quality	0.31	0.01	1.57	−2.78	3.39	0.844
Social Network – Diversity	−0.45	−0.04	0.83	−2.08	1.18	0.589
Social Network – Number of people	0.27	0.15	0.13	0.01	0.52	0.042
Fatigue (FSS)	4.66	0.43	0.72	3.25	6.08	<0.001*
DASS-21 Depression 2020	−1.41	−0.30	0.41	−2.20	−0.60	0.001*
DASS-21 Anxiety 2020	−0.95	−0.15	0.48	−1.90	0.01	0.051
DASS-21 Stress 2020	−1.12	−0.28	0.37	−1.86	−0.39	0.003*

SE, standard error; CI, confidence interval; *LL*, lower limit; *UL*, upper limit. *R*^2^ = 0.44, *F*(17, 222) = 10.24, *p* < 0.001. *Statistically significant.

### 3.3. Association between emotional burden and SARS-CoV-2 infection

Data did not provide proof of an association between higher depression, anxiety, and stress levels (DASS-21) in seronegative subjects in 2020 and confirmed SARS-CoV-2-infection in 2021. In contrast, the odds of people with a self-reported diagnosis of mental disorder in 2020 for a future infection with SARS-CoV-2 in 2021 was almost four times higher than that of people without any prior self-reported mental disorder. Moreover, subjects reporting a higher number of sleep hours had lower odds for SARS-CoV-2 infection (see Table 8).

**Table 8:** Binary logistic regression analyses showing the association between depression, anxiety and stress (DASS-21) in 2020 and confirmed SARS-CoV-2-infection in 2021 (*n* = 305).

Variables	OR	SE	95%CI		*p*
			*LL*	*UL*	
DASS-21 Depression 2020	0.99	0.09	0.83	1.18	0.896
DASS-21 Anxiety 2020	0.94	0.10	0.77	1.14	0.521
DASS-21 Stress 2020	0.98	0.08	0.84	1.13	0.737
Age (in years)	0.96	0.01	0.94	0.99	0.009*
Sex (reference: male)	1.07	0.38	0.51	2.25	0.864
Native tongue (reference: German)	1.21	0.25	0.74	1.97	0.451
Employment	0.71	0.21	0.47	1.08	0.112
Educational level (reference: below high school)	0.76	0.36	0.38	1.55	0.455
Vaccinated against COVID-19 (reference: yes)	1.81	0.36	0.88	3.69	0.105
One or more chronic diseases (reference: no disease)	1.37	0.96	0.21	9.02	0.746
Diagnosed mental disorder (reference: no mental disorder)	3.75	0.38	1.79	7.83	<0.001*
Sleep – Hours	0.68	0.18	0.48	0.96	0.028*
Sleep - Quality	1.38	0.35	0.69	2.73	0.360
Social Network - Diversity	0.79	0.19	0.55	1.13	0.198
Social Network - Number of people	1.03	0.03	0.97	1.09	0.313
Fatigue (FSS)	1.22	0.15	0.92	1.63	0.171

OR, odds ratio; SE, standard error; CI, confidence interval; *LL*, lower limit; *UL*, upper limit. *Statistically significant.

## 4. Discussion

This longitudinal study revealed a significant decrease in depression and stress levels (as measured by the DASS-21) in the general population of South Tyrol (Northern Italy) between the coronavirus pandemic in 2020 and 2021. Moreover, a previous SARS-CoV-2 infection resulted in a negative impact on emotional burden (i.e., depression, anxiety, and stress levels). The risk of developing relevant depressive symptoms after infection was more than three times higher than that in non-infected subjects. While the emotional burden itself, as measured with the DASS-21, was not an explanatory factor for infection with SARS-CoV-2, subjects with a self-reported diagnosed mental disorder were more likely to be infected prospectively.

The COVID-19 outbreak in 2020, with shocking images of army trucks transporting coronavirus victims in Lombardy (Italy), the implementation of transmission and protection measures (i.e., lockdown, social distancing), together with a fear of contracting COVID-19, uncertainty of the future, and financial instability, had a negative impact on the population’s emotional burden (WHO, 2022), especially among younger adults and women (Kunzler et al., 2021; Ma et al., 2021; Prati and Mancini, 2021; Racine et al., 2021; Schelhorn et al., 2021; Robinson et al., 2022). A systematic review by Santomauro et al. (2021) estimated a global increase in major depressive disorder by 27.6% and anxiety disorders by 25.6% due to the COVID-19 pandemic. Similar to previous longitudinal survey studies (Mata et al., 2021), we observed patterns of habituation over time with decreasing depression and stress and stable anxiety levels. Although we did not observe an association between vaccination and SARS-CoV-2 infection or emotional burden in our data, the decrease in depression and stress levels might be related to the introduction of safe and effective vaccines against COVID-19., as a national study of U.S. adults revealed (Koltai et al., 2022).

The findings from our study confirm the elevated risk of developing new and relevant depressive, anxiety, and stress symptoms after a serologically confirmed infection with SARS-CoV-2, even after controlling for potentially confounding variables such as fatigue, and support to the hypothesis of a psycho-neuroendocrine-immune interplay in COVID-19. Both a (sub)chronic inflammatory response and cytokine storm might be involved in the immediate manifestation of neuropsychiatric symptoms in individuals with COVID-19 infections (Debnath et al., 2020). Previous clinical research on long COVID or post COVID conditions described the various long-term effects and mental health issues in both hospitalized patients with severe COVID-19 illness (Michelen et al., 2021; Chen et al., 2022; Houben-Wilke et al., 2022) and non-hospitalized patients with mild clinical illness (Graham et al., 2021; Han et al., 2022). In addition, psychological mechanism, such as personality (e.g., lower emotional stability) and dysfunctional coping strategies (i.e., COVID anxiety syndrome) could have a moderating effect of an infection with SARS-CoV-2 on psychological symptoms (Nikčević and Spada, 2020; Mansueto et al., 2021). However, the negative impact of SARS-CoV-2 infection on mental health should therefore be highlighted more clearly in COVID-19 campaigns to inform the general population. Individuals after COVID-19, especially those with previous mental health issues, could profit from a systematic screening for mental burden from healthcare professionals (e.g., general practitioners, nurses) to identify those individuals in need for psychological and psychiatric support, e.g., stress-reducing interventions to improve resilience, with the objective to improve mental health and to reduce the likelihood for developing mental disorders after a SARS-CoV-2 infection.

Although depression, anxiety, and stress levels in 2020 were not predictive for an infection with SARS-CoV-2 within the following 12 months, our analyses revealed that people with a diagnosed mental disorder were more likely to be infected with SARS-CoV-2. This is in contrast to a recent WHO policy brief stating that there is no consistent evidence that people living with mental disorders are more susceptible to COVID-19 (WHO, 2022). While one meta-analysis found an increased risk of COVID-19 in people with pre-existing mood disorders, anxiety, and attention-deficit hyperactivity disorder, another meta-analysis comparing people with and without pre-existing mood disorders found no evidence of increased susceptibility to COVID-19 (Ceban et al., 2021; Liu et al., 2021). Further longitudinal research is necessary to explore whether and what types of pre-existing mental disorders are independent risk factors for SARS-CoV-2 infection, more severe COVID-19 and subsequent negative outcomes, including hospitalization and mortality, while including psychological variables like personality, coping stile and resilience as possible moderating variables. To reduce the risk of developing severe illness or complications from COVID-19, healthcare providers should advise and support individuals with pre-existing mental health conditions to take extra precautions to reduce their risk of exposure to SARS-CoV-2, as well as to improve health behaviors to strengthen the immune system (e.g., nutrition, physical activity and stress reduction measures) (Ingram et al., 2020; Fazia et al., 2022).

Interestingly, our data showed that higher sleep duration was associated with lower odds for SARS-CoV-2 infection. Sleep is a key factor in maintaining overall health and immunity and sleep disorders are a common feature of mental health problems that have been more frequently observed during the COVID-19 pandemic. Evidence suggests that obstructive sleep apnea is an independent risk factor for severe COVID-19 (Strausz et al., 2021). While sleep problems were found to be associated with higher levels of psychological distress during the COVID-19 pandemic (Alimoradi et al., 2021), we did not observe an association between sleep quality and duration and emotional burden in our study. Given that COVID-19 is a respiratory illness that can cause severe illness and complications, it is important to understand the role of sleep in the immune response to the virus. Further investigations are required to explore whether our finding might be related to specific shift-working professions that suffer from sleep deprivation and are therefore at higher risk for infection (e.g., healthcare professionals).

### 4.1. Strengths and limitations

The strengths of our study are the longitudinal design and the selection of a representative sample from the general population. Moreover, an infection with SARS-CoV-2 was confirmed using a SARS-CoV-2-specific immunoglobulin blood test and did not rely on self-reports. This study has several limitations that must be considered. As the participation/response rate in both data collections was below the expected 40–50% (Guo et al., 2016), the findings need to be interpreted in light of a possible participation bias. Although most participants reporting increased depression, anxiety and stress levels at the second data collection (June–July 2021) were most likely infected during the second and third waves in fall/winter 2020/2021, we were unable to determine the exact timing of the infection and therefore to prove the chronicity of emotional burden after an infection with SARS-CoV-2. Given the time period of this longitudinal study, the findings and conclusions are limited to infections with SARS-CoV-2 variants alpha and delta, although recent research found similar neurological and psychiatric outcomes during the delta and omicron waves (Taquet et al., 2022). As diagnosed mental disorders were self-reported, we cannot exclude that participants’ responses were affected by the pandemic (e.g., political situation, pandemic management) leading to an over- or underreporting.

## 5. Conclusion

This longitudinal study included a sample from the general population of South Tyrol (Northern Italy) participating in two data collections during the coronavirus pandemic in 2020 and 2021. Data confirmed the negative impact of SARS-CoV-2 infection on depression, anxiety, and stress levels. While emotional burden itself was not associated with an infection with SARS-CoV-2, persons with a previously diagnosed mental disorder were more likely to be infected. Psychological and psychiatric support, for example, stress-reducing interventions, may improve the resilience and immune defense of individuals with diagnosed mental disorders and help restore mental health after SARS-CoV-2 infection. Further research is necessary to explore whether and (what types of) mental disorders are independent risk factors for SARS-CoV-2 infection, more severe COVID-19 and subsequent negative outcomes, including hospitalization and mortality.

## Data availability statement

Data will be made available from the corresponding author upon request.

## Ethics statement

The studies involving human participants were reviewed and approved by Ethics committee of Bolzano on 28/05/2020 (approval number 52-2020) and the amendment on 22/04/21 (approval number 51-2021). Written informed consent to participate in this study was provided by the participants’ legal guardian/next of kin.

## Author contributions

AM, AE, GP, MM, HM, RK, and DA developed the idea for the study. SL, TG, CW, AM, AE, GP, MM, HM, RK, GeP, PS, FG, and DA contributed to the concept, design, and data collection. GeP, PS, FG, RP, SL, TG, MM, DA, AM, AE, GP, and RK contributed to the analysis and interpretation of the data. DA, CW, and RK contributed to the drafting of the manuscript. All authors contributed to the critical revision of the manuscript and approved the final version.

## Funding

The study was funded by the local representatives of the National Health Service (Azienda Sanitaria dell’Alto Adige/Südtiroler Sanitätsbetrieb – ASDAA/SABES).

## Conflict of interest

The authors declare that the research was conducted in the absence of any commercial or financial relationships that could be construed as a potential conflict of interest.

## Publisher’s note

All claims expressed in this article are solely those of the authors and do not necessarily represent those of their affiliated organizations, or those of the publisher, the editors and the reviewers. Any product that may be evaluated in this article, or claim that may be made by its manufacturer, is not guaranteed or endorsed by the publisher.

## Footnotes

^1^www.soscisurvey.com

^2^https://diagnostics.roche.com/global/en/products/params/elecsys-anti-sars-cov-2.html, accessed June 19, 2022.
